# Long-term exposures to ambient particulate matter and ozone pollution with lower extremity deep vein thrombosis after surgical operations: a retrospective case-control study in Beijing, China

**DOI:** 10.1186/s12889-023-16882-3

**Published:** 2023-10-09

**Authors:** Qin Xiong, Wanzhou Wang, Yong Wang, Min Zhang, Benqiang Rao, Xuezhao Ji, Zhihu Xu, Shaowei Wu, Furong Deng

**Affiliations:** 1 Education Department, Beijing Shijitan Hospital, Capital Medical University, Beijing, 100038 China; 2 Department of Occupational and Environmental Health Sciences, School of Public Health, Peking University, Beijing, 100191 China; 3 National Institute of Health Data Science, Peking University Health Science Center, Beijing, China; 4 Beijing First Aid Center, Beijing, 100031 China; 5 Medical Insurance Management Office, Beijing Shijitan Hospital, Capital Medical University, Beijing, 100038 China; 6 Second Ward of Gastrointestinal Surgery, Beijing Shijitan Hospital, Capital Medical University, Beijing, 100038 China; 7 Department of Occupational and Environmental Health, School of Public Health, Xi’an Jiaotong University Health Science Center, Xi’an, Shaanxi China

**Keywords:** Ambient air pollution, Fine particulate matter, Inhalable particulate matter, Ozone, Venous thromboembolism, Deep vein thrombosis

## Abstract

**Background:**

Lower extremity deep vein thrombosis (LEDVT) after surgical operations is a common and fatal disease leading to unfavorable outcomes including death. Nevertheless, there has been insufficient evidence on the associations between ambient air pollution and LEDVT, particularly studies from developing regions.

**Methods:**

Based on 302 LEDVT cases and 302 controls in a general hospital in Beijing, China, this unmatched retrospective case-control study investigated the associations of fine particulate matter (PM_2.5_), inhalable particulate matter (PM_10_), and ozone (O_3_) with odds of LEDVT.

**Results:**

Per 10 μg/m^3^ increase in PM_2.5_, PM_10_, and O_3_ at 3-month, 6-month, and 2-year average was associated with increased LEDVT odds [odds ratios (ORs) for PM_2.5_: 1.10 (95%CI: 1.05, 1.14), 1.14 (95%CI: 1.09, 1.18), and 1.30 (95%CI: 1.06, 1.61); ORs for PM_10_: 1.06 (95%CI: 1.02, 1.10), 1.12 (95%CI: 1.08, 1.16), and 1.29 (95%CI: 1.03, 1.61); ORs for O_3_: 1.00 (95%CI: 0.96, 1.04), 1.16 (95%CI: 1.02, 1.31), and 2.08 (95%CI: 1.03, 4.18), respectively]. The stratified analyses, exposure-responses curves, and sensitivity analyses further highlighted the robustness of our findings.

**Conclusions:**

Long-term exposures to ambient PM_2.5_, PM_10_, and O_3_ may increase the risk of LEDVT in patients after surgical operations. The results may be implicated in the prevention and control of adverse clinical outcomes of surgical patients associated with ambient air pollution.

**Supplementary Information:**

The online version contains supplementary material available at 10.1186/s12889-023-16882-3.

## Introduction

An increasing number of studies have linked ambient air pollution, particularly fine particulate matter (PM_2.5_), inhalable particulate matter (PM_10_), and ozone (O_3_) to elevated all-cause mortality and cardiovascular disease risks and mortality across the globe [1–4]. The global burden of disease (GBD) estimated that exposures to PM_2.5_ and O_3_ contributed to 4.2 million and 0.25 million deaths, and 103.1 million and 4.1 million disability-adjusted life-years (DALYs) in 2015, respectively [5]. Specifically, people in low-and-middle-income countries (LMICs) are facing elevated disease burden attributable to the geographical context of higher air pollution concentrations [5].

Deep vein thrombosis (DVT) is a common disease leading to unfavorable outcomes including stroke, acute coronary syndromes, and deaths [6–8]. The vast majority of DVT cases develop in the legs, which is widely recognized as lower extremity deep vein thrombosis (LEDVT) [8]. The LEDVT is a common and fatal disease that frequently occurs in patients after surgical operations, leading to swelling, pain, and possibly pulmonary embolism (PE) and death [8]. Significantly, according to the International Society on Thrombosis and Haemostasis (ISTH), a considerable proportion (about 1/3 ~ 1/2) of the venous thromboembolism (VTE) cases lack identifiable causative factors [9]. Identifying the provoking factors of DVT would be beneficial for the prevention of adverse outcomes after surgical operations.

Several studies have linked ambient air pollution to elevated risks of vascular diseases especially DVT [6, 10–13]. The proinflammatory and prothrombotic effects of air pollutants may be underlying driving factors. Exposures to PM_2.5_, PM_10_, and O_3_ are associated with increased levels of inflammatory and coagulatory factors [14, 15], and may lead to elevated risks of DVT triggered by blood coagulation system [16, 17]. Nevertheless, most of the current epidemiological studies were conducted in developed regions (e.g. the US and Italy) with relatively low ambient air pollution concentrations. The insufficient empirical evidence may not be robust enough to draw a firm conclusion. In light of this, this study investigated the associations between long-term exposures to PM_2.5_, PM_10_, and O_3_ and the odds of LEDVT. Our results may be implicated in the prevention and control of disease burden associated with ambient air pollution.

## Methods

### Study participants

**Fig. 1:**
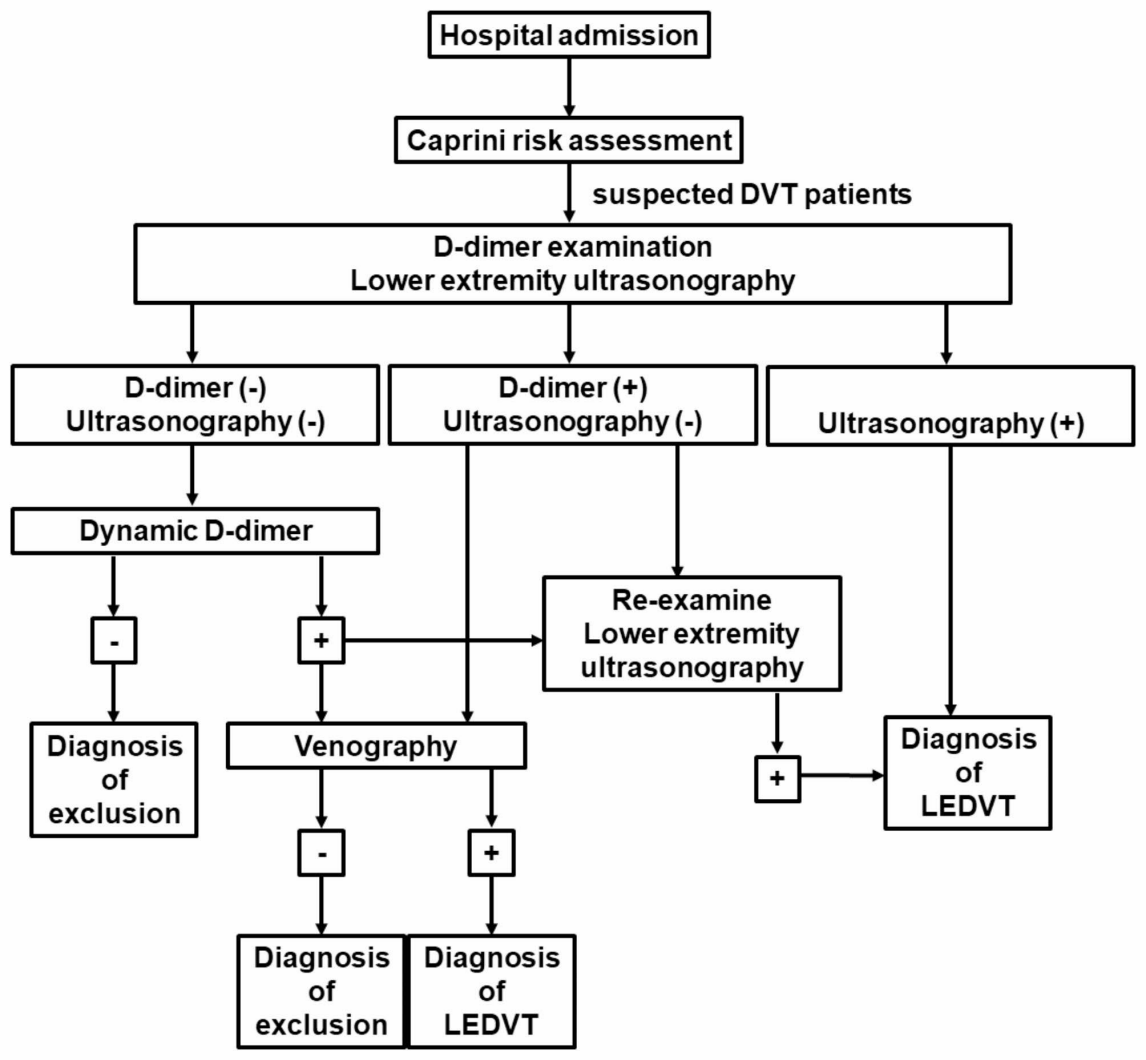
Flowchart for the clinical diagnosis of LEDVT

The participants of this unmatched retrospective case-control study were retrieved from the administrative medical records of Beijing Shijitan Hospital, Capital Medical University. The cases and controls were all surgical hospitalized patients from March 2017 to March 2020. The cases were (a) patients diagnosed with LEDVT by lower extremity ultrasonography after surgical operations and (b) without a history of diagnosed VTE, including DVT or PE previously. The exclusion criteria were: (a) patients with the initial admission diagnosis of VTE and (b) recurrent VTE within 3 months. The controls were randomly selected from (a) surgical patients after surgical operations, (b) without a diagnosis of VTE confirmed by lower extremity ultrasonography, and (c) without a history of diagnosed VTE. The detailed standard clinical procedures for the diagnosis of LEDVT in this study are presented in Fig. 1. In total, 302 cases and 302 controls were finally included.

We gathered information on patients’ sex, age, day of hospital admission, day of surgical operation, residential address, body mass (obesity, defined as ≥ 28.0 kg/m^2^ according to the guidelines for the Chinese population [18, 19], or not), history of diabetes, history of cardiovascular diseases (CVDs), and intensive care unit stay. In this study, each hospitalized participant received a Caprini risk assessment within 24 h of hospitalization by trained physicians [7]. The Caprini risk assessment is a widely acknowledged effective tool to evaluate the risks of LEDVT based on a series of standard items with specific scores and has been widely used in previous studies [20, 21]. The risk levels of participants were categorized into highest, high, moderate, and low risks according to the Caprini risk scores [7].

This study has been approved by the Institutional Review Board of Beijing Shijitan Hospital [record number: sjtkyll-lx-2020(62)]. The data without personal identifiers were gathered for administrative management of DVT patients after surgical operations, and thus the study was exempted from informed consent.

### Exposure assessments

Data on daily ambient air pollutants (PM_2.5_, PM_10_, and maximum 8 h average O_3_) were collected from the National Urban Air Quality Real-Time Release Platform (online accessible websites: http://106.37.208.233:20035/). Meanwhile, data on meteorological variables (including temperature and relative humidity) were gathered from the China Meteorological Administration (online accessible websites: http://data.cma.cn/) [22]. In addition, we also collected data from provincial administrative environmental release platforms to fill in the missing data (< 0.5%) [23]. The environment exposures to PM_2.5_, PM_10_, O_3_, temperature, and humidity were generated based on the nearest administrative fixed-site monitoring stations (all the distances were less than 10 km). The same government quality control standards are used to operate all the ambient air pollution monitoring stations in China, which could assure the consistency of PM_2.5_, PM_10_ and O_3_ data from different monitoring stations [24].

To characterize the associations between long-term exposures to PM_2.5_, PM_10_, and O_3_ and odds of LEDVT, this study generated 3-month average, 6-month average, and 2-year average concentrations preceding the day of surgical operation for each participant. The exposure windows were selected based on previous publications [6, 11, 12, 25].

### Statistical analyses

Generalized linear models with a “logit” link function (logistic regression models) were used to estimate the associations between long-term exposures to PM_2.5_, PM_10_, and O_3_ and the odds of LEDVT of the participants. The formula can be expressed as:

$$logit\left(\text{y}\right)={{b}_{0}+\beta exposure+b}_{1}{x}_{1}+\cdots +{b}_{n}{x}_{n}+\epsilon$$

where the outcome variable y is a binary variable (LEDVT case or control), the b_0_ is the intercept, the b_1_ × _1_…b_n_x_n_ denote the covariates and their regression coefficients, and the ε denotes the residuals. In the main effect models, we included individual’s Caprini risk level (highest, high, moderate, or low risks) as a covariate [7]. Meanwhile, in order to control for the temporal variations in weeks and seasons, we included a day-of-week (category) variable and a month (category) variable in the models [26]. Meanwhile, we also included the temperature and relative humidity at the same exposure windows with PM_2.5_, PM_10_, or O_3_ to exclude the potential confounding effects of meteorological conditions.

To evaluate the potential differences in the effect estimates between population subgroups, this study conducted stratified analyses according to participants’ sex (female or male) and age (< 75 years or ≥ 75 years). The *Z*-test statistics were used to examine the differences in the effect estimates between the two groups, which were calculated based on the regression coefficients and the standard errors of the variable of interest (PM_2.5_, PM_10_, or O_3_) [1, 27].

Several sensitivity analyses were conducted to examine the robustness of our results. First, we plotted the exposure-response curves for the associations between air pollutants and odds of LEDVT. This method would demonstrate the odds of LEDVT along with the increment in pollutant concentration across the exposure ranges [1]. Following the methods reported previously, the linear effect term of the pollutant variable was replaced with a cubic spline function with three degrees of freedom [1]. Second, we conducted two-pollutant models by adding the co-exposure pollutant at the same exposure windows in the models. Third, we included multiple covariates, including age, sex (female or male), obesity (yes or no), diabetes (yes or no), CVDs (yes or no), D-dimer levels (≥ 500 ng/mL or < 500 ng/mL), staying in the intensive care unit (yes or no), day-of-week (category), month (category), temperature, and relative humidity instead of the Caprini risk levels in the models [6, 12, 13, 16, 28]. Fourth, we generated average pollutant concentrations over 3-month, 6-month, and 2-year preceding the admission day of each participant as the exposure. Fifth, to address the potential non-linear confounding effects of temperature [29], we replaced the linear term with a natural cubic spline function with 3 degrees of freedom. The results of regression analyses were reported as estimated odds ratio (OR) and its 95% confidence intervals (95%CIs) of LEDVT per 10 μg/m^3^ in pollutant concentration.

All the statistical analyses were conducted using the R software (version 3.6.3) incorporated with the “splines” and “nlme” packages. A two-sided *P* < 0.05 was defined as statistical significance.

## Results

### Study participants

As shown in Table 1, a total of 302 LEDVT cases (170 females and 132 males) and 302 controls (134 females and 168 males) were included in this unmatched retrospective case-control study, with an average age of 70.1 years and 58.3 years, respectively. Of the 302 cases, 24 (7.9%) participants were obese and 56 (18.5%) participants had a history of diabetes. Meanwhile, of the 302 controls, 33 (10.9%) participants were obese and 48 (15.9%) participants had a history of diabetes. The Caprini risk assessment indicated that 181 (59.9%), 118 (39.1%), 3 (1.0%), and 0 (0.0%) cases and 56 (18.5%), 94 (31.1%), 63 (20.9%), and 89 (29.5%) controls were with highest, high, moderate, and low risks of LEDVT, respectively.

**Table 1:** Baseline characteristics of the study participants

Characteristic	Total (n = 604)	Case (n = 302)	Control (n = 302)	*P*-value
Age	64.2 ± 7.3	70.1 ± 12.9	58.3 ± 19.0	< 0.001
Sex				0.004
Female	304 (50.3%)	170 (56.3%)	134 (44.4%)	
Male	300 (49.7%)	132 (43.7%)	168 (55.6%)	
Obesity (≥ 28 kg/m^2^)	57 (9.4%)	24 (7.9%)	33 (10.9%)	0.266
Diabetes	104 (17.2%)	56 (18.5%)	48 (15.9%)	0.451
CVDs	48 (7.9%)	35 (11.6%)	13 (4.3%)	0.004
Intensive care unit	90 (14.9%)	55 (18.2%)	35 (11.6%)	0.030
D-dimer				< 0.001
≥ 500 ng/mL	299 (49.5%)	222 (73.5%)	77 (25.5%)	
< 500 ng/mL	149 (24.7%)	61 (20.2%)	88 (29.1%)	
Not examined	156 (25.8%)	19 (6.3%)	137 (45.4%)	
Caprini risk				< 0.001
Highest risk	237 (39.2%)	181 (59.9%)	56 (18.5%)	
High risk	212 (35.1%)	118 (39.1%)	94 (31.1%)	
Moderate risk	66 (10.9%)	3 (1.0%)	63 (20.9%)	
Low risk	89 (14.7%)	0 (0.0%)	89 (29.5%)	

Note: Data are shown as mean ± standard deviation or number (proportion). Chi-square test and student t test were used to examine the subgroup differences

### Air pollution and meteorological exposures

The distributions of ambient PM_2.5_, PM_10_, O_3_, temperature, and relative humidity exposures of the participants are presented in Table 2. Generally, we observed higher average concentrations of PM_2.5_, PM_10_, and O_3_ for cases compared to controls at the selected exposure windows.

Generally, there were significant positive correlations between PM_2.5_ and PM_10_, and significant negative associations between PM and O_3_ (Table S1).

**Table 2:** Distributions of ambient PM_2.5_, PM_10_, O_3_, temperature, and relative humidity exposures of the participants

Variable	Total (n = 604)	Case (n = 302)	Control (n = 302)	*P*-value
**3-month average**				
PM_2.5_	53.3 ± 10.5	53.5 ± 8.6	53.0 ± 12.0	0.565
PM_10_	81.3 ± 13.0	82.6 ± 12.6	80.0 ± 13.3	0.014
O_3_	98.8 ± 43.0	104.9 ± 40.3	92.7 ± 44.7	< 0.001
Temperature	13.7 ± 9.6	14.9 ± 9.2	12.5 ± 10.0	0.002
Relative humidity	49.0 ± 11.1	49.4 ± 10.6	48.6 ± 11.7	0.360
**6-month average**				
PM_2.5_	57.9 ± 15.0	59.8 ± 17.3	56.0 ± 12.1	0.002
PM_10_	86.1 ± 15.7	87.5 ± 18.0	84.7 ± 12.9	0.029
O_3_	97.5 ± 32.1	98.4 ± 30.6	96.5 ± 33.6	0.462
Temperature	13.3 ± 7.1	13.7 ± 7.0	12.9 ± 7.2	0.181
Relative humidity	49.9 ± 6.9	51.4 ± 6.0	48.5 ± 7.3	< 0.001
**2-year average**				
PM_2.5_	63.3 ± 10.6	65.8 ± 10.1	60.8 ± 10.5	< 0.001
PM_10_	91.2 ± 10.5	93.7 ± 9.9	88.8 ± 10.5	< 0.001
O_3_	98.9 ± 3.1	99.3 ± 3.0	98.6 ± 3.1	0.002
Temperature	14.1 ± 0.3	14.2 ± 0.2	14.0 ± 0.3	< 0.001
Relative humidity	51.5 ± 2.0	51.8 ± 2.1	51.2 ± 1.8	0.001

Note: Data are shown as mean ± standard deviation. The student t test was used to examine the subgroup differences

### Associations between long-term exposures to PM_2.5_, PM_10_, and O_3_ with odds of LEDVT

Long-term exposures to PM_2.5_, PM_10_, and O_3_ were associated with increased odds of LEDVT at selected exposure windows (Table 3). Per 10 μg/m^3^ increase in PM_2.5_ exposure concentration at 3-month, 6-month, and 2-year average was associated with increased LEDVT odds, with ORs of 1.10 (95%CI: 1.05, 1.14), 1.14 (95%CI: 1.09, 1.18), and 1.30 (95%CI: 1.06, 1.61), respectively. Each 10 μg/m^3^ increase in PM_10_ exposure concentration at 3-month, 6-month, and 2-year average was associated with ORs of 1.06 (95%CI: 1.02, 1.10), 1.12 (95%CI: 1.08, 1.16), and 1.29 (95%CI: 1.03, 1.61) of LEDVT. In addition, a 10 μg/m^3^ increase in O_3_ exposure concentration at 6-month and 2-year average was associated with increased LEDVT odds, with ORs of 1.16 (95%CI: 1.02, 1.31) and 2.08 (95%CI: 1.03, 4.18), respectively.

Stratified analyses indicated that there were no significant differences in the effect estimates between female and male participants or older and younger participants (Table S2).

### Sensitivity analyses

**Fig. 2:**
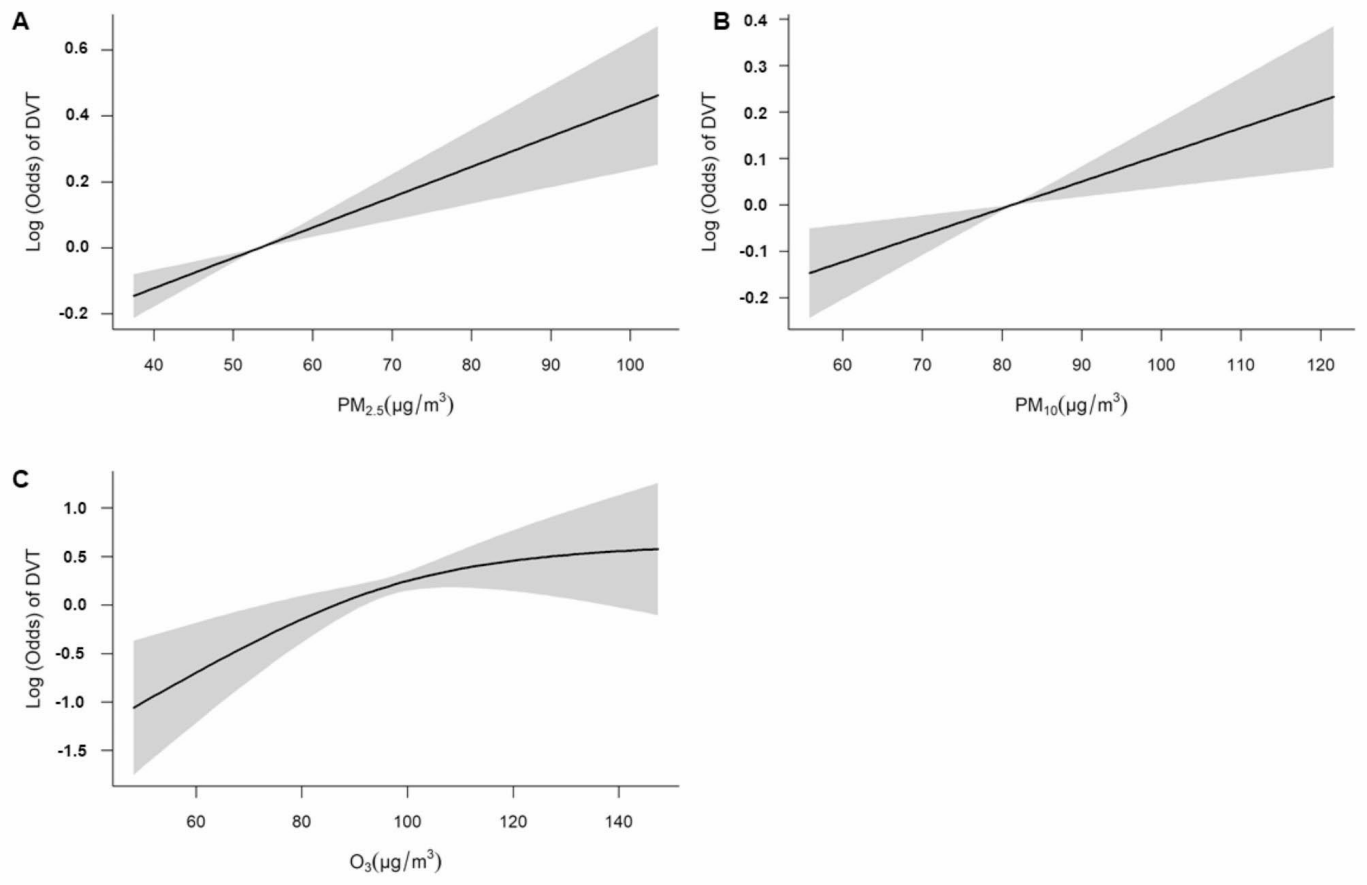
Exposure-response curves for the associations between PM_2.5_, PM_10_, and O_3_ with odds of LEDVT. Note: The models were adjusted for Caprini risk, day-of-week, month, temperature, and relative humidity

The exposure-response curves demonstrated generally linear associations between PM_2.5_, PM_10_, and O_3_ and odds of LEDVT over the exposure ranges, as shown in Fig. 2. Results of two-pollutant models indicated that after controlling for co-pollutant, the associations between PM_2.5_, PM_10_, and O_3_ and LEDVT were generally stable (Table S3). In addition, models controlling for multiple covariates and using exposures preceding the hospital admission day further highlighted the robustness of the results, as shown in Table S4. After replacing the linear temperature term with the non-linear spline function, the results were also stable (Table S4).

**Table 3:** Associations between long-term exposures to PM_2.5_, PM_10_, and O_3_ with odds of LEDVT

Exposure	Effect estimates
**3-month average**	
PM_2.5_	1.10 (1.05, 1.14)
PM_10_	1.06 (1.02, 1.10)
O_3_	1.00 (0.96, 1.04)
**6-month average**	
PM_2.5_	1.14 (1.09, 1.18)
PM_10_	1.12 (1.08, 1.16)
O_3_	1.16 (1.02, 1.31)
**2-year average**	
PM_2.5_	1.30 (1.06, 1.61)
PM_10_	1.29 (1.03, 1.61)
O_3_	2.08 (1.03, 4.18)

Note: Results were shown as estimated ORs (95% CI) of LEDVT associated with a 10 μg/m^3^ increase in the air pollutant concentration. The models were adjusted for Caprini risk, day-of-week, month, temperature, and relative humidity

## Discussion

Identifying the environmental risk factors of LEDVT would be beneficial for the management of unfavorable outcomes after surgical operations. The present unmatched retrospective case-control study based on patients after surgical operations indicated that long-term exposures to major ambient air pollutants, including PM_2.5_, PM_10_, and O_3_ were associated with increased odds of LEDVT. Meanwhile, the stratified analyses, exposure-responses curves, and sensitivity analyses further strengthen the robustness of our findings.

Several previous studies have linked long-term ambient air pollution exposures to increased risks of DVT. A cohort study among Italians observed increased DVT risks along with the increment in long-term PM_2.5_ exposure concentration [12]. A case-crossover study among the elderly in the US found that long-term exposure to PM_2.5_ was associated with increased risks of DVT admissions [13]. A case-control study found that long-term exposure to PM_10_ was associated with an increase in DVT risk among people in Italy [10]. In addition, a cohort study among the middle-aged population from the Atherosclerosis Risk in Communities Study in the US found that people with higher residential traffic density had higher risks of developing DVT [11]. Meanwhile, another case-control study found that living near major traffic roads was also associated with increased risks of DVT among people in Italy [6]. Nevertheless, current evidence was generally developed regions with relatively low air pollution concentrations. In this study, we observed constantly increased odds of LEDVT along with the increment in PM_2.5_, PM_10_, and O_3_ exposure concentrations. Our results may thus serve as beneficial evidence in developing countries with the background of severe air quality.

Our study found similar associations between ambient air pollution exposure and odds of LEDVT among female and male participants, and participants < 75 years and ≥ 75 years. The aforementioned two studies in Italy observed stronger associations in male compared to female participants, and insignificant differences among participants in different age groups [6, 10]. Nevertheless, one study in Italy found stronger associations in younger participants compared to the older [12]. Meanwhile, one study in Italy and one study in the US reported insignificant differences between female and male participants [12, 13]. The differences may be partially attributable to the heterogeneity in study design, population characteristics, and geographical background. Nevertheless, current evidence is still insufficient to draw a firm conclusion.

The underlying biological mechanisms for the associations between ambient air pollution and LEDVT have not been fully clarified. One probable hypothesis is the proinflammatory and prothrombotic effects of air pollutants [17, 30, 31]. Previous meta-analyses reported that exposures to PM_2.5_, PM_10_, and O_3_ were associated with increased levels of inflammation and coagulation factors [14, 15]. Accordingly, long-term exposures to air pollutants may result in DVT through activation of blood coagulation system [16, 17, 32].

To our best knowledge, this study provides the first empirical evidence on the associations between ambient air pollution and LEDVT in Asian-Pacific regions. The relatively higher air pollution concentrations in Beijing, China would also provide beneficial evidence for regions with relatively higher air pollution concentrations. However, several limitations should be declared. First, the study was subjected to the Berkson’s bias due to the nature of the unmatched retrospective case-control design, which may result in a loss of statistical power [33]. However, the participants of our study were limited to patients after surgical operations, and excluded participants with a history of VTE, which to some extent reduced the heterogeneity between the subjects [7]. Second, this study did not collect information on physical activity of the participants, which may potentially confound the associations between air pollution and LEDVT. Previous studies indicate that physical activity can attenuate the negative effects of air pollution on the cardiovascular system [34, 35], nevertheless, this not addressed in this study. Third, the fixed-site environmental monitoring data may not characterize the individual exposure levels to the air pollutants, which may lead to exposure misclassifications resulting in under-estimation of the exposure-health associations. Fourth, considering the differences between medical and surgical patients, our findings should be generalized cautiously to the general hospitalized individuals.

## Conclusions

In conclusion, long-term exposures to ambient PM_2.5_, PM_10_, and O_3_ may increase the risk of LEDVT of patients after surgical operations. The results may be implicated in the prevention and control of adverse clinical outcomes of surgical patients. Meanwhile, our study also indicated the importance of controlling the health effects associated with ambient air pollution at the population level.

### Electronic supplementary material

Below is the link to the electronic supplementary material.

Supplementary Material 1

## Data Availability

The datasets used and/or analysed during the current study are available from the corresponding author on reasonable request.

## Abbreviations

CVDs: Cardiovascular diseases
DALYs: Disability-adjusted life-years
DVT: Deep vein thrombosis
GBD: Global burden of disease
ISTH: The International Society on Thrombosis and Haemostasis
LEDVT: Lower extremity deep vein thrombosis
LMICs: Low-and-middle-income countries
O_3_: Ozone
PE: Pulmonary embolism
PM_10_: Inhalable particulate matter
PM_2.5_: Fine particulate matter
VTE: Venous thromboembolism
