# Supplementary material for: Long-term exposures to ambient particulate matter and ozone pollution with lower extremity deep vein thrombosis after surgical operations: a retrospective case-control study in Beijing, China
Source: BMC Public Health. 2023 Oct 9;23:1956. doi: 10.1186/s12889-023-16882-3 (PMC10563341; doi:10.1186/s12889-023-16882-3)
Supplement: Supplementary file 1 — Supplementary Material 1 [file 12889_2023_16882_MOESM1_ESM.docx]

**Long-term exposures to ambient particulate matter and ozone pollution with lower extremity deep vein thrombosis after surgical operations: A case-control study in Beijing, China**

Qin Xiong ^1,#,*^, Wanzhou Wang ^1,2,3,#^, Yong Wang ^4^, Min Zhang ^5^, Benqiang Rao ^6^, Xuezhao Ji ^1,2^, Zhihu Xu ^1,2^, Shaowei Wu ^7^, Furong Deng ^2,*^

**Affiliations:**

^1^ Education Department, Beijing Shijitan Hospital, Capital Medical University, Beijing 100038, China

^2^ Department of Occupational and Environmental Health Sciences, School of Public Health, Peking University, Beijing 100191, China

^3^ National Institute of Health Data Science, Peking University Health Science Center, Beijing, China

^4^ Beijing First Aid Center, Beijing 100031, China

^5^ Medical Insurance Management Office, Beijing Shijitan Hospital, Capital Medical University, Beijing 100038, China

^6^ Second Ward of Gastrointestinal Surgery, Beijing Shijitan Hospital, Capital Medical University, Beijing 100038, China

^7^ Department of Occupational and Environmental Health, School of Public Health, Xi’an Jiaotong University Health Science Center, Xi’an, Shaanxi, China

^#^ QX and WW are joint-first authors.

*** Correspondence:**

Qin Xiong, PhD, Education Department, Beijing Shijitan Hospital, Capital Medical University, Beijing 100038, China. Email: [xiongqinsmile@163.com](mailto:xiongqinsmile@163.com)

Furong Deng, MD, PhD, Department of Occupational and Environmental Health Sciences, School of Public Health, Peking University, Beijing 100191, China, E-mail: [lotus321321@126.com](mailto:lotus321321@126.com)

**Table S1.** Spearman correlation coefficients of the ambient air pollution and meteorological variables.

**Table S2.** Associations between long-term exposures to PM_2.5_, PM_10_, and O_3_ with odds of LEDVT, stratified by sex and age.

**Table S3.** Associations between long-term exposures to PM_2.5_, PM_10_, and O_3_ with odds of LEDVT in the two-pollutant models.

**Table S4.** Associations between long-term exposures to PM_2.5_, PM_10_, and O_3_ with odds of LEDVT in the sensitivity analyses.

**Table S1.** Spearman correlation coefficients of the ambient air pollution and meteorological variables.

|  | **PM_2.5_** | **PM_10_** | **O_3_** | **Temperature** | **Relative humidity** |
| --- | --- | --- | --- | --- | --- |
| **3-month average** |  |  |  |  |  |
| PM_2.5_ | — | 0.84* | 0.01 | -0.12* | -0.22* |
| PM_10_ |  | — | 0.25* | 0.04 | -0.28* |
| O_3_ |  |  | — | 0.92* | 0.51* |
| Temperature |  |  |  | — | 0.75* |
| Relative humidity |  |  |  |  | — |
| **6-month average** |  |  |  |  |  |
| PM_2.5_ | — | 0.89* | -0.33* | -0.45* | -0.54* |
| PM_10_ |  | — | -0.12* | -0.24* | -0.48* |
| O_3_ |  |  | — | 0.95* | 0.48* |
| Temperature |  |  |  | — | 0.70* |
| Relative humidity |  |  |  |  | — |
| **2-year average** |  |  |  |  |  |
| PM_2.5_ | — | 0.96* | -0.35* | -0.14* | 0.74* |
| PM_10_ |  | — | -0.31* | 0.01 | 0.71* |
| O_3_ |  |  | — | 0.04 | -0.69* |
| Temperature |  |  |  | — | -0.26* |
| Relative humidity |  |  |  |  | — |

***** *P*<0.05.

**Table S2.** Associations between long-term exposures to PM_2.5_, PM_10_, and O_3_ with odds of LEDVT, stratified by sex and age.

| **Group** | **3-month** | **6-month** | **2-year** |
| --- | --- | --- | --- |
| **PM_2.5_** |  |  |  |
| **Sex** |  |  |  |
| Female | 1.09 (1.02, 1.18) | 1.18 (1.09, 1.28) | 1.27 (0.84, 1.94) |
| Male | 1.11 (1.05, 1.18) | 1.15 (1.09, 1.22) | 1.39 (1.06, 1.84) |
| *P*-value | 0.701 | 0.610 | 0.729 |
| **Age** |  |  |  |
| age <75 years | 1.12 (1.06, 1.19) | 1.15 (1.08, 1.22) | 1.38 (1.03, 1.83) |
| age ≥75 years | 1.06 (1.00, 1.13) | 1.13 (1.06, 1.20) | 1.25 (0.90, 1.74) |
| *P*-value | 0.217 | 0.662 | 0.676 |
| **PM_10_** |  |  |  |
| **Sex** |  |  |  |
| Female | 1.04 (0.98, 1.10) | 1.16 (1.08, 1.24) | 1.18 (0.78, 1.78) |
| Male | 1.09 (1.03, 1.15) | 1.13 (1.07, 1.18) | 1.40 (1.04, 1.89) |
| *P*-value | 0.245 | 0.543 | 0.501 |
| **Age** |  |  |  |
| age <75 years | 1.09 (1.03, 1.14) | 1.14 (1.08, 1.20) | 1.39 (1.03, 1.90) |
| age ≥75 years | 1.02 (0.96, 1.08) | 1.10 (1.04, 1.16) | 1.20 (0.85, 1.69) |
| *P*-value | 0.108 | 0.399 | 0.527 |
| **O_3_** |  |  |  |
| **Sex** |  |  |  |
| Female | 1.00 (0.93, 1.07) | 1.21 (0.97, 1.52) | 1.57 (0.44, 5.63) |
| Male | 0.97 (0.92, 1.03) | 1.11 (0.94, 1.31) | 2.97 (1.11, 7.95) |
| *P*-value | 0.626 | 0.524 | 0.441 |
| **Age** |  |  |  |
| age <75 years | 1.01 (0.96, 1.07) | 1.25 (1.04, 1.49) | 2.28 (0.91, 5.67) |
| age ≥75 years | 1.00 (0.94, 1.06) | 1.03 (0.84, 1.27) | 1.71 (0.56, 5.18) |
| *P*-value | 0.785 | 0.177 | 0.695 |

Note: Results were shown as estimated ORs (95% CI) of LEDVT associated with a 10 μg/m^3^ increase in the air pollutant concentration. The models were adjusted for Caprini risk, day-of-week, month, temperature, and relative humidity.

**Table S3.** Associations between long-term exposures to PM_2.5_, PM_10_, and O_3_ with odds of LEDVT in the two-pollutant models.

| **Exposure** | **Main effect** | **Pollutant-PM_2.5_** | **Pollutant-PM_10_** | **Pollutant-O_3_** |
| --- | --- | --- | --- | --- |
| **3-month average** |  |  |  |  |
| PM_2.5_ | 1.10 (1.05, 1.14) | — | 1.24 (1.12, 1.37) | 1.10 (1.05, 1.14) |
| PM_10_ | 1.06 (1.02, 1.10) | 1.13 (1.04, 1.24) | — | 1.06 (1.02, 1.10) |
| O_3_ | 1.00 (0.96, 1.04) | 0.99 (0.95, 1.03) | 1.01 (0.97, 1.05) | — |
| **6-month average** |  |  |  |  |
| PM_2.5_ | 1.14 (1.09, 1.18) | — | 1.22 (0.95, 1.56) | 1.14 (1.09, 1.19) |
| PM_10_ | 1.12 (1.08, 1.16) | 1.06 (0.85, 1.33) | — | 1.12 (1.08, 1.17) |
| O_3_ | 1.16 (1.02, 1.31) | 0.98 (0.84, 1.15) | 1.00 (0.86, 1.17) | — |
| **2-year average** |  |  |  |  |
| PM_2.5_ | 1.30 (1.06, 1.61) | — | 2.14 (0.82, 5.56) | 1.25 (1.00, 1.55) |
| PM_10_ | 1.29 (1.03, 1.61) | 1.72 (0.63, 4.72) | — | 1.21 (0.95, 1.54) |
| O_3_ | 2.08 (1.03, 4.18) | 1.74 (0.80, 3.80) | 1.70 (0.78, 3.73) | — |

Note: Results were shown as estimated ORs (95% CI) of LEDVT associated with a 10 μg/m^3^ increase in the air pollutant concentration. The models were adjusted for Caprini risk, day-of-week, month, temperature, and relative humidity. Pollutant-PM_2.5_, additionally adjusted for PM_2.5_. Pollutant-PM_10_, additionally adjusted for PM_10_. Pollutant-O_3_, additionally adjusted for O_3_.

**Table S4.** Associations between long-term exposures to PM_2.5_, PM_10_, and O_3_ with odds of LEDVT in the sensitivity analyses.

| **Exposure** | **Main effect*^a^*** | **Model S1*^b^*** | **Model S2*^c^*** | **Model S3*^d^*** | **Model S4*^e^*** |
| --- | --- | --- | --- | --- | --- |
| **3-month average** |  |  |  |  |  |
| PM_2.5_ | 1.10 (1.05, 1.14) | 1.10 (1.06, 1.14) | 1.09 (1.05, 1.13) | 1.10 (1.06, 1.14) | 1.12 (1.06 ,1.17) |
| PM_10_ | 1.06 (1.02, 1.10) | 1.07 (1.03, 1.11) | 1.06 (1.02, 1.10) | 1.07 (1.03, 1.11) | 1.07 (1.02 ,1.11) |
| O_3_ | 1.00 (0.96, 1.04) | 0.99 (0.96, 1.03) | 1.03 (0.99, 1.07) | 1.03 (0.99, 1.07) | 0.99 (0.95 ,1.04) |
| **6-month average** |  |  |  |  |  |
| PM_2.5_ | 1.14 (1.09, 1.18) | 1.14 (1.09, 1.18) | 1.13 (1.08, 1.17) | 1.12 (1.08, 1.17) | 1.14 (1.09 ,1.20) |
| PM_10_ | 1.12 (1.08, 1.16) | 1.12 (1.08, 1.16) | 1.11 (1.07, 1.15) | 1.10 (1.06, 1.14) | 1.13 (1.08 ,1.17) |
| O_3_ | 1.16 (1.02, 1.31) | 1.12 (0.99, 1.26) | 1.20 (1.05, 1.36) | 1.19 (1.05, 1.36) | 1.12 (0.98 ,1.27) |
| **2-year average** |  |  |  |  |  |
| PM_2.5_ | 1.30 (1.06, 1.61) | 1.32 (1.08, 1.62) | 1.63 (1.28, 2.09) | 1.61 (1.26, 2.05) | 1.31 (1.06 ,1.61) |
| PM_10_ | 1.29 (1.03, 1.61) | 1.31 (1.06, 1.62) | 1.56 (1.22, 1.99) | 1.52 (1.19, 1.93) | 1.29 (1.04 ,1.60) |
| O_3_ | 2.08 (1.03, 4.18) | 1.80 (0.95, 3.41) | 1.71 (0.88, 3.32) | 1.58 (0.82, 3.04) | 2.12 (1.05 ,4.30) |

Note: Results were shown as estimated ORs (95% CI) of LEDVT associated with a 10 μg/m^3^ increase in the air pollutant concentration.

*^a^* The models were adjusted for Caprini risk, day-of-week, month, temperature, and relative humidity.

*^b^* The models were adjusted for age, sex, obesity, diabetes, CVDs, D-dimer levels, staying in intensive care unit, day-of-week, month, temperature, and relative humidity.

*^c^* The exposure concentrations were generated based on exposures preceding the day of hospital admissions. The models were adjusted for Caprini risk, day-of-week, month, temperature, and relative humidity.

*^d^* The exposure concentrations were generated based on exposures preceding the day of hospital admissions. The models were adjusted for age, sex, obesity, diabetes, CVDs, D-dimer levels, staying in intensive care unit, day-of-week, month, temperature, and relative humidity.

*^e^* The models were adjusted for Caprini risk, day-of-week, month, temperature, and relative humidity. The linear temperature term was replaced with a natural cubic spline function with 3 degrees of freedom.
